# Molecular Description of a Novel *Orientia* Species Causing Scrub Typhus in Chile

**DOI:** 10.3201/eid2609.200918

**Published:** 2020-09

**Authors:** Katia Abarca, Constanza Martínez-Valdebenito, Jenniffer Angulo, Ju Jiang, Christina M. Farris, Allen L. Richards, Gerardo Acosta-Jamett, Thomas Weitzel

**Affiliations:** Escuela de Medicina, Pontificia Universidad Católica de Chile, Santiago, Chile (K. Abarca, C. Martínez-Valdebenito, J. Angulo);; Naval Medical Research Center, Silver Spring, Maryland, USA (J. Jiang, C.M. Farris);; Uniformed Services University of the Health Sciences, Bethesda, Maryland, USA (A.L. Richards);; Facultad de Ciencias Veterinarias, Universidad Austral de Chile, Valdivia, Chile (G. Acosta-Jamett);; Clínica Alemana de Santiago, Facultad de Medicina Clínica Alemana, Universidad del Desarrollo, Santiago (T. Weitzel)

**Keywords:** Bacteria Candidatus Orientia chiloensis, Candidatus Orientia chuto, Chile, epidemiology, genus-specific quantitative PCR, molecular sequence data, Orientia tsutsugamushi, rickettsia, Rickettsiales, scrub typhus, trombiculid mites, tsutsugamushi triangle

## Abstract

Scrub typhus is a potentially fatal rickettsiosis caused by *Orientia* species intracellular bacteria of the genus *Orientia*. Although considered to be restricted to the Asia Pacific region, scrub typhus has recently been discovered in southern Chile. We analyzed *Orientia* gene sequences of 16S rRNA (*rrs*) and 47-kDa (*htrA*) from 18 scrub typhus patients from Chile. Sequences were ≥99.7% identical among the samples for both amplified genes. Their diversity was 3.1%–3.5% for *rrs* and 11.2%–11.8% for *htrA* compared with *O. tsusugamushi* and 3.0% for *rrs* and 14.8% for *htrA* compared with *Candidatus* Orientia chuto. Phylogenetic analyses of both genes grouped the specimens from Chile in a different clade from other *Orientia* species. Our results indicate that *Orientia* isolates from Chile constitute a novel species, which, until they are cultivated and fully characterized, we propose to designate as *Candidatus* Orientia chiloensis, after the Chiloé Archipelago where the pathogen was identified.

Scrub typhus is a potentially fatal rickettsial infection transmitted by larval stage trombiculid mites called chiggers. Scrub typhus is caused by *Orientia tsutsugamushi*, a strictly intracellular bacterium with a remarkable genetic and antigenic diversity (*1*,*2*). Although this disease has been known since at least 313 ce and currently threatens over 1 billion people in Asia and Australasia, it is widely underdiagnosed and underreported (*3*,*4*). Disinterest has been influenced by the perception that scrub typhus is a geographically limited disease, threatening rural populations within a certain region, known as the “tsutsugamushi triangle,” and rarely affecting travelers (*5*,*6*). 

This paradigm, however, has recently been brought into question with evidence of scrub typhus being found in the Middle East, Africa, and South America (*7*–*12*). Genomic information on *Orientia* strains from Chile has been insufficient and scrub typhus in the Middle East region is caused by a new *Orientia* species, *Candidatus* Orientia chuto (*6*), highlighting that our current knowledge on the spectrum of *Orientia* species is incomplete (*13*). Here, we discuss the molecular description and phylogenetic analysis of a potential third pathogenic *Orientia* species detected in 18 patients with scrub typhus in southern Chile. 

## Materials and Methods

### Patients and Samples

The clinical samples described in this study were derived from 18 patients with confirmed scrub typhus diagnosed during February 2016–February 2019. All cases were acquired in southern Chile and diagnosed as part of an ongoing surveillance project of the Chilean Rickettsia and Zoonosis Research Group. The project was approved by the Comité Ético Científico, Pontificia Universidad Católica de Chile (Santiago, Chile; #12–170 and #160816007) and the Naval Medical Research Center (Silver Spring, MD, USA; PJT-16-24) (*9*,*14*,*15*). 

We collected, stored, and extracted DNA from buffy coat preparations and eschar specimens as described elsewhere (*9*,*15*). Eschar samples consisted of swab specimens taken from the base or crust material of eschar, which we mechanically desegregated using sterile glass beads. DNA was automatically extracted from eschar and blood samples using MagNA Pure System (Roche Molecular Systems, https://diagnostics.roche.com), according to the manufacturer’s instructions. All included DNA specimens derived from eschar samples, except the BM2016-I sample, which was only from the buffy coat specimen. 

### PCR Assays and Sequencing 

We initially assessed all extracted DNA samples by a newly designed genus-specific quantitative PCR (Orien16S qPCR assay), as described elsewhere (*15*,*16*). For further analysis, we performed seminested PCRs targeting the 16S rRNA gene (*rrs*), 47-kDa high-temperature requirement A gene (*htrA*), and 56-kDa type-specific antigen gene (*tsa*) to the qPCR Orien16S–positive samples (Appendix Table 1). We sequenced PCR amplicons for both DNA strands using Sanger sequencing method (Psomagen Inc., https://psomagen.com). Two independent investigators analyzed the chromatogram of each sequence and aligned them using BioEdit version 7.0.5.3 (*17*) Sequences from these scrub typhus patients in Chile were submitted to GenBank under accession no. MK329247 (*rrs*), MN231837 (*rrs*), MT435057 (*rrs*), MK343091 (*htrA*), and MT431624 (*htrA*). 

### Phylogenetic Analysis 

We compared *Orientia* DNA sequences from the 2016–2019 scrub typhus patients with DNA from the first *Orientia* scrub typhus patient in Chile from 2006 and that of distinct *Orientia* species, including *O. tsutsugamushi* and *Candidatus* O. chuto, different *Rickettsia* species, and other microorganisms retrieved from GenBank and aligned using ClustalW (http://www.clustal.org). We used MEGAX software (https://www.megasoftware.net) to infer phylogenetic analyses by the maximum-likelihood method (*18*) and to perform the search for the most appropriate model of nucleotide substitution for phylogenetic analysis according to the Bayesian information criterion. For the maximum-likelihood method, we obtained initial trees for the heuristic search automatically by applying neighbor-joining and Bio neighbor-joining algorithms to a matrix of pairwise distances estimated using the maximum composite likelihood approach and then selecting the topology with superior log likelihood value. We based the support of the topology on a bootstrapping of 1,000 replicates; the positions equivalent to gaps or missing data were deleted. 

### Comparison of Nucleotide Diversity

We created consensus sequences of the generated amplicons after alignment in BioEdit version 7.0.5.3, which we compared with respective sequences of *O. tsutsugamushi* and *Candidatus* O. chuto strains as well as the first *Orientia* case from Chile, obtained from GenBank. A sequence identity matrix was constructed in BioEdit version 7.0.5.3. The selected databases and algorithms used for alignment and comparison of sequences were in accordance with current recommendations for the taxonomical characterization of prokaryote strains (*19*). 

## Results

### Cases

The 18 investigated scrub typhus cases were acquired in 3 regions currently known to be endemic for scrub typhus (*15*), Biobío, Los Lagos, and Aysen, which span >1,120 km (latitude 38°03¢S to 47°47¢S) in Chile; 5 of the 18 cases were from Chiloé Island (Los Lagos), where the initial case was reported (Table 1). At the time they sought treatment, all but 1 patient exhibited the 3 clinical signs characteristic of scrub typhus: fever, maculopapular rash, and inoculation eschar. The presence of *Orientia* genomic DNA was confirmed in all cases by qPCR Orien16S from buffy coat or eschar material (Table 1). All of the patients recovered from scrub typhus without sequelae, 16 after treatment with doxycycline, 1 after treatment with azithromycin, and 1 without specific antimicrobial therapy. Further epidemiologic and clinical details of some of the patients have been published elsewhere (*9*,*15*,*20*). 

**Table 1:** Epidemiologic, clinical, and diagnostic features of 18 scrub typhus patients, southern Chile, 2016–2019*

Patient no.	Isolate no.	Patient age, y/sex	Exposure			Clinical signs				qPCR results		Ref.
			Date	Region		Fever	Rash	Eschar		Buffy coat	Eschar	
1	BM2016-I	55/M	2016 Feb	Los Lagos†		Yes	Yes	Yes		Positive	ND	(*9*)
2	LC2016-I	42/M	2016 Feb	Los Lagos†		Yes	No	Yes		ND	Positive	(*30*)
3	MS2016-M	43/M	2016 Mar	Aysén		Yes	Yes	Yes		Positive	Positive	(*15*)
4	IS2017-M	56/M	2017 Feb	Los Lagos		Yes	Yes	Yes		Positive	Positive	(*15*)
5	NV2017-I	73/M	2017 Feb	Los Lagos†		Yes	Yes	Yes		ND	Positive	(*30*)
6	AE2018-M	25/M	2018 Feb	Los Lagos		Yes	Yes	Yes		ND	Positive	(*15*)
7	FC2018-M	22/F	2018 Feb	Los Lagos		Yes	Yes	Yes		Positive	Positive	(*15*)
8	AF2018-M	39/M	2018 Feb	Los Lagos		Yes	Yes	Yes		Positive	Positive	(*15*)
9	EC2018-M	28/M	2018 Feb	Biobío		Yes	Yes	Yes		Positive	Positive	(*15*)
10	VP2018-M	21/F	2018 Mar	Los Lagos		Yes	Yes	Yes		Positive	Positive	(*15*)
11	SH2018-M	49/F	2018 Dec	Los Lagos		Yes	Yes	Yes		Negative	Positive	(*30*)
12	GM2019-I	30/F	2019 Feb	Los Lagos†		Yes	Yes	Yes		ND	Positive	(*30*)
13	JC2019-I	54/M	2019 Feb	Los Lagos†		Yes	Yes	Yes		ND	Positive	(*30*)
14	CC2019-M	63/M	2019 Feb	Los Lagos		Yes	Yes	Yes		ND	Positive	(*30*)
15	CV2019-M	23/F	2019 Feb	Los Lagos		Yes	Yes	Yes		ND	Positive	(*30*)
16	MA2019-M	53/M	2019 Feb	Los Lagos		Yes	Yes	Yes		ND	Positive	(*30*)
17	MV2019-M	54/F	2019 Feb	Los Lagos		Yes	Yes	Yes		ND	Positive	(*30*)
18	SG2019-M	41/M	2019 Feb	Los Lagos		Yes	Yes	Yes		ND	Positive	(*30*)

*ND, not done; ref., reference.  †Chiloé Island.

### DNA Sequences and Phylogenetic Analyses

**Figure 1:**
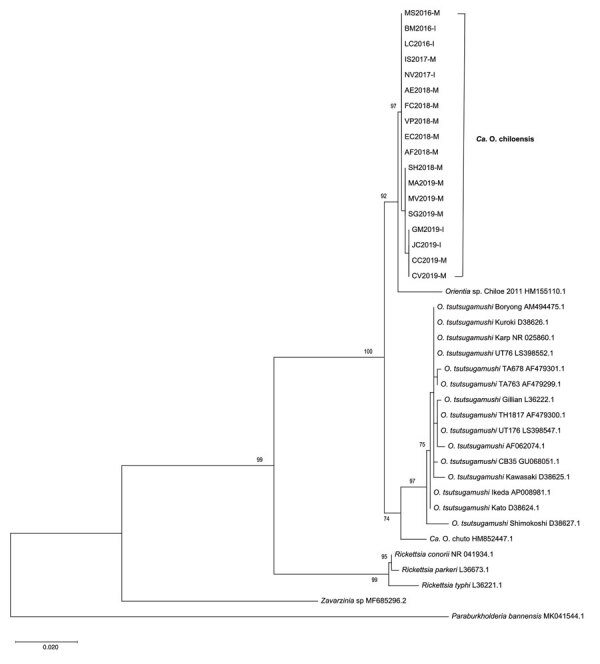
Phylogenetic analyses of sequences of the 16S rRNA gene (*rrs*) from scrub typhus cases in Chile compared with those from different *Orientia* and *Rickettsia* species and other microorganisms. We inferred the evolutionary history by using the maximum-likelihood method based on the Kimura 2-parameter model (*21*), according to the Bayesian information criterion for these sequences. The analysis involved 39 nt sequences and a total of 875 positions in the final dataset. The trees is drawn to scale, with branch lengths measured in the number of substitutions per site. All positions containing gaps and missing data were eliminated. For isolates from the cases in this study, the suffix “M” indicates an origin in mainland Chile and “I” an origin on Chiloé Island; these isolates clustered into a proposed new species provisionally named *Candidatus* Orientia chiloensis. GenBank accession numbers are indicated for reference sequences. Scale bar indicates nucleotide divergence.

**Figure 2:**
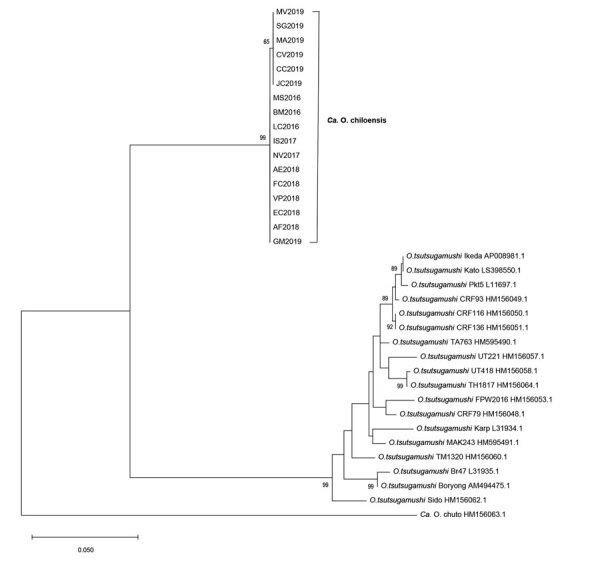
Phylogenetic analyses of sequences of the 47-kDa gene (*htrA*) from scrub typhus cases from Chile in comparison to different *Orientia* species. For the phylogenetic tree, the maximum-likelihood method based on the Hasegawa-Kishino-Yano model was applied (*22*). A discrete gamma distribution was used to model evolutionary rates differences among sites. The analysis involved 37 nucleotide sequences and a total of 736 positions in the final dataset. The tree is drawn to scale, with branch lengths measured in the number of substitutions per site. All positions containing gaps and missing data were eliminated. Isolates from the cases in this study clustered into a proposed new species provisionally named *Candidatus* Orientia chiloensis. GenBank accession numbers are indicated for reference sequences. Scale bar indicates nucleotide divergence.

We successfully amplified fragments of *rrs* from 18 cases and *htrA* from17 cases; the primers for *tsa* failed to produce amplicons. For all assays, we successfully amplified a well-defined *Orientia* strain (Kawasaki clade) from South Korea as a positive control (*6*). The lengths of clean reads were 886 nt for *rrs* and 950 nt for *htrA*. Sequences of the isolates showed a high nucleotide identity (99.7%–100%) for both genes (Appendix Table 1), with a maximum divergence of 2 nucleotides. We were able to distinguish 3 distinct *rrs* genotypes (1, 2, and 3) and 2 genotypic variants of *htrA* (a and b) (Appendix Table 2). *HtrA* variants, although determined by only 1 nucleotide, led to distinct DNA codons with leucine versus phenylalanine. The genotype 1a samples (n = 10) derived from Los Lagos (continental and Chiloé Island), Biobío, and Aysén regions, whereas genotypes 2b (n = 3) occurred in the continental Los Lagos region, 3a (n = 1) in Chiloé Island, and genotype 3b (n = 3) in the Los Lagos region, both continental and Chiloé Island. For 1 genotype 2 strain, we could not amplify *htrA* (Appendix Table 2). Phylogenetic analyses of both genes from the DNA specimens from Chile formed a unique cluster separate from the 14 *O. tsutsugamushi* strains included in the analysis as well as from *Candidatus* O. chuto (Figures 1, 2). However, the *rrs* sequence from the 2016–2019 samples grouped together with that from the first scrub typhus case in Chile (Figure 1) (*8*). 

Details of discrepancies in the nucleotide sequences were evaluated by identity matrices. For *rrs* from the isolates from Chile, identity of the consensus sequence ranged from 96.5% to 97.0% compared with *O. tsutsugamushi* and *Candidatus* O. chuto (Table 2). A higher diversity was observed for *htrA*, with sequence identity of 88.2%–88.8% compared with *O. tsutsugamushi* and 85.2% with *Candidatus* O. chuto (Table 3). We observed a GTA insertion (valine) in position 28 of *htrA* in all 18 samples, similar to *Candidatus* O. chuto, but this substitution was not observed in *O. tsutsugamushi* strains. 

**Table 2:** Identity matrix of 16S rRNA gene (*rrs*) showing percentage of pairwise identity of 886 nt consensus sequence of 18 recent *Candidatus* Orientia chiloensis cases in Chile with the first *Orientia* case from Chile in 2006, 16 *O. tsutsugamushi* strains, and *Candidatus* Orientia chuto

Sequences*†	1	2	3	4	5	6	7	8	9	10	11	12	13	14	15	16	17
1 Chiloensis																	
2 Chiloe 2006	98.0																
3 Chuto	97.0	95.8															
4 Boryong	96.7	95.7	97.7														
5 Kuroki	96.7	95.7	97.7	100													
6 Gilliam	96.5	95.4	97.5	99.7	99.7												
7 Karp	96.8	95.8	97.8	99.8	99.8	99.6											
8 Kawasaki	96.7	95.7	97.1	99.2	99.2	99.2	99.3										
9 Ikeda	96.9	95.9	97.9	99.7	99.7	99.5	99.8	99.2									
10 Kato	96.9	95.9	97.9	99.7	99.7	99.5	99.8	99.2	100								
11 Shimokoshi	96.9	96.1	96.9	98.4	98.4	98.4	98.5	98.6	98.6	98.6							
12 CB35	96.7	95.7	97.9	99.7	99.7	99.5	99.8	99.2	99.7	99.7	98.4						
13 TA678	96.6	95.6	97.6	99.6	99.6	99.4	99.7	99.0	99.6	99.6	98.3	99.6					
14 TA763	96.5	95.4	97.5	99.5	99.5	99.3	99.6	98.9	99.5	99.5	98.1	99.5	99.6				
15 TH1817	96.5	95.6	97.5	99.5	99.5	99.5	99.6	98.9	99.5	99.5	98.1	99.5	99.4	99.3			
16 UT176	96.8	95.8	97.6	99.6	99.6	99.6	99.7	99.3	99.6	99.6	98.5	99.6	99.5	99.4	99.6		
17 UT76	96.8	95.8	97.8	99.8	99.8	99.6	100	99.3	99.8	99.8	98.5	99.8	99.7	99.6	99.6	99.7	
18 Litchfield	96.6	95.6	97.1	99.2	99.2	99.2	99.3	98.9	99.2	99.2	98.5	99.2	99.0	98.9	99.2	99.5	99.3
*All sequences from strains of *O.* *tsutsugamushi* except 1 consensus sequence *Candidatus* Orientia chiloensis; 2 *Orientia* species; and 3 *Candidatus* Orientia chuto. †Numbers in the column headers (top row) indicate the same sequences as those associated with the same numbers in the first column. GenBank accession numbers: 2, HM 15510.1; 3, HM852447.1; 4, AM494475.1; 5, D38626.1; 6, L36222.1; 7, NR_025860.1; 8, D38625.1; 9, AP008981.1; 10, D38624.1; 11, D38627.1; 12, GU068051.1; 13, AF479301.1; 14, AF479299.1; 15, AF479300.1; 16, LS398547.1; 17, LS398552.1; 18, AF062074.1.																	

**Table 3:** Identity matrix of 47-kDa high temperature requirement A gene (*htrA*) showing percentage of pairwise identity of 950 nt consensus sequence of 17 *Candidatus* Orientia chiloensis cases from Chile with 18 strains of *O. tsutsugamushi* and *Candidatus* Orientia chuto

Sequences*†	1	2	3	4	5	6	7	8	9	10	11	12	13	14	15	16	17	18	19
1 Chiloensis																			
2 Chuto	85.2																		
3 Ikeda	88.5	83.7																	
4 Pkt5	88.3	83.5	99.7																
5 Kp47	88.2	83.7	97.5	97.3															
6 Br47	88.3	83.8	96.9	96.7	96.5														
7 Boryong	88.4	83.7	97.2	97.0	96.8	99.4													
8 CRF116	88.3	83.5	99.5	99.3	97.5	96.9	97.2												
9 FPW2016	88.3	83.5	98.0	97.8	97.5	97.1	97.6	98.0											
10 Kato	88.5	83.7	100	99.7	97.5	96.9	97.2	99.5	98.0										
11 MAK243	88.8	84.2	98.5	98.3	98.0	97.5	97.8	98.5	98.0	98.5									
12 CRF79	88.3	83.7	98.7	98.5	97.7	96.9	97.2	98.7	98.7	98.7	98.5								
13 TM1320	88.6	83.4	98.4	98.2	97.0	97.4	97.7	98.2	97.8	98.4	97.9	97.6							
14 UT221	88.8	84.1	98.5	98.3	96.9	97.1	97.3	98.5	97.4	98.5	98.2	98.0	97.5						
15 UT418	88.4	84.1	98.5	98.3	97.5	96.9	97.2	98.5	97.5	98.5	98.2	98.4	97.4	98.4					
16 Sido	88.7	83.6	96.9	96.7	96.0	96.7	97.2	96.7	97.3	96.9	97.2	96.7	97.2	96.9	96.3				
17 CRF93	88.5	83.7	99.6	99.4	97.5	96.9	97.2	99.6	98.3	99.6	98.5	98.7	98.2	98.4	98.6	96.9			
18 CRF136	88.3	83.5	99.5	99.3	97.5	96.9	97.2	100	98.0	99.5	98.5	98.7	98.2	98.5	98.5	96.7	99.6		
19 TA763	88.6	83.8	99.1	98.9	97.8	97.1	97.6	99.1	98.3	99.1	98.7	98.9	97.7	98.5	98.7	97.0	99.0	99.1	
20 TH1817	88.3	84.0	98.5	98.3	97.4	96.8	97.1	98.5	97.4	98.5	98.0	98.3	97.3	98.4	99.7	96.1	98.4	98.5	98.7
*All sequences from strains of *O.* *tsutsugamushi* except 1 consensus sequence *Candidatus* Orientia chiloensis; 2 *Orientia* species; and 3 *Candidatus* Orientia chuto. †Numbers in the column headers (top row) indicate the same sequences as those associated with the same numbers in the first column. GenBank accession numbers: 2, HM156063.1; 3, AP008981.1; 4, L11697.1; 5, L31934.1; 6, L31935.1; 7, AM494475.1; 8, HM156050.1; 9, HM156053.1; 10, LS398550.1; 11, HM595491.1; 12, HM156048.1; 13, HM156060.1; 14, HM156057.1; 15, HM156058.1; 16, HM156062.1; 17, HM156049.1; 18, HM156051.1; 19, HM595490.1; 20, HM156064.1.																			

### Species Designation

The *rrs* sequences we analyzed showed a divergence of ≥3% from known *Orientia* species, indicating that the isolates from Chile constitute a novel species within the genus *Orientia* (family *Rickettsiaceae*, order Rickettsiales, class Alphaproteobacteria). Our designation of the bacteria as a new species was corroborated by the divergence of *htrA* and our inability to generate amplicons with primers of *O. tsutsugamushi* type-specific antigen gene *tsa*. Until a type strain is cultivated and characterized, we propose the designation *Candidatus* Orientia chiloensis for the novel species, after the Chiloé Archipelago (Los Lagos Region, Chile) where the pathogen was first identified (*8*,*9*). 

## Discussion 

Because of new diagnostic tools and increasing clinical awareness, our knowledge of rickettsial infections has increased over recent decades (*23*,*24*). For scrub typhus, which has been considered the most important rickettsiosis in Asia and Australasia, the discovery of new endemic regions outside of the traditional tsutsugamushi triangle raises questions about established paradigms (*25*). Since 2006, multiple patients with scrub typhus have been reported in southern Chile, >12,000 km away from known endemic regions (*8*,*9*,*15*). In addition, a case of scrub typhus caused by a novel species, *Candidatus* O. chuto, was diagnosed on the Arabian Peninsula (*7*). These findings, together with serologic and molecular data from sub-Saharan Africa and Europe, suggest that scrub typhus caused by various *Orientia* species might have a much wider than previously known, possibly global, distribution (*4*,*26*,*27*).

Most clinicoepidemiologic and ecologic aspects of scrub typhus in South America are currently unknown. A recent study on Chiloé Island suggested that trombiculid mites of the genus *Herpetacarus*, which were found to be infected with *Orientia*-species bacteria, might serve as vectors (*28*); preliminary phylogenetic analyses showed that the mite-associated strains were 99%–100% identical to those from patients (*29*). Clinically, the >40 patients with scrub typhus diagnosed in southern Chile during 2015–2019 sought treatment for conditions similar to those for scrub typhus from the Asia Pacific region—fever, generalized rash, and inoculation eschar—and, similarly, had a rapid response to treatment with tetracycline or azithromycin (*30*). Early molecular and serologic data suggest that the *Orientia* species in Chile diverge from those in the Asia-Pacific region (*8*,*9*,*15*), but whether they represent distinct *O. tsutsugamushi* strains or a new species remained inconclusive. Our phylogenetic analyses of larger DNA segments from 2 conserved genes support the conclusion that the isolates from patients in Chile cluster outside known *Orientia* species and represent a distinct species. 

Culture-independent sequencing techniques play an important role in prokaryotic taxonomy, especially for strictly intracellular bacteria (*31*,*32*). For the description of new species, sequence analyses of the 16S rRNA gene (*rrs*) are paramount. A ≥3% divergence of *rrs* sequences from those in known species is the accepted threshold suggesting a novel species (*19*), although corrected levels of ≥1.30%–1.35% have been suggested (*33*,*34*). Isolates with *rrs* sequence differences of >5%–6% might belong to a distinct genus, if they display unique phenotypic differences (*35*). Distinct, lower thresholds have been developed for *Rickettsia* spp. (*31*), but this approach remains controversial among rickettsiologists (*36*). As should be the case for all molecularly defined novel species and genera, we have classified this proposed species as *Candidatus*, until type strains can be cultivated and fully described (*37*). 

The novel *Orientia* species presented here fulfills the *rrs* gene criteria described in the previous sections. Our designation of a novel species was affirmed by a high divergence of another genomic marker, *htrA*, which diverged >11% from *O. tsutsugamushi* and of >14% from *Candidatus* O. chuto (Table 3). This conserved gene diverges only <3.7% among *O. tsutsugamushi* isolates (*38*). The *O. tsutsugamushi* type-specific antigen gene, *tsa*, which has a much higher diversity than *htrA* (*1*), was not amplifiable from isolates from Chile using primers designed for *O. tsutsugamushi*. This suggests that the *Candidatus* O. chiloensis *tsa* is unique, requiring the assessment of additional primers, possibly based on results from a future WGS. Currently, no *Orientia* culture isolate from Chile is available. 

Surprisingly, the *rrs* and *htrA* sequences from the 18 *Orientia* samples from Chile were almost identical, showing a maximum variability of only 2 nucleotides. This genetic homogeneity over a wide geographic range is in sharp contrast to *O. tsutsugamushi* (*38*). As a unique characteristic among obligate intracellular pathogens, this species displays a dramatic genomic and phenotypic heterogeneity (*1*), which might be related to homologous recombination and lateral gene transfer (*39*). Among *O. tsutsugamushi* isolates, for example, the divergence of reported *rrs* sequences are up to 1.5% and for *htrA* sequences up to 3.6% (*1*,*38*), compared with ≤0.3% observed among isolates from *Orientia* DNA from Chile. 

The most frequently applied phenotypic and molecular marker of *O. tsutsugamushi* strain heterogeneity is the highly variable 56-kDa TSA. This *Orientia*-specific surface protein is also known to be an important determinant of strain-specific pathogenicity and immunity (*1*). As we mentioned, we were not able to generate amplicons of strains from Chile with the applied *tsa* PCR or with *tsa* qPCR (*41*) or other commonly used primers (e.g., r56_2057). In a previous report, short *tsa* sequences were produced from 2 samples, but only after prolonged amplification cycles (*9*). These findings strongly suggest that *tsa* of *Candidatus* O. chiloensis is highly divergent from those of other *Orientia* species. Because the TSA surface protein is the main antigenic determinant, such divergence might explain the low serologic cross-reactivity, which was observed in patients with scrub typhus and in seroprevalence studies in Chile using *O. tsutsugamushi* whole-cell or recombinant antigens (*9*,*41*). 

In conclusion, our results indicate that scrub typhus in Chile is caused by a novel *Orientia* species, suggesting an ancient origin of the disease in South America, rather than recent introduction. However, after obtaining cultured isolates of *Candidatus* O. chiloensis and larger gene sequences including from WGS, deeper comparative studies of the 3 *Orientia* species and their vectors are necessary to understand the ecology and evolution of these emerging intracellular pathogens, including the mechanisms responsible for the differences in strain variability and surface proteins. 

Appendix Additional information for molecular description of a novel *Orientia* species causing scrub typhus in Chile.
