# Supplementary material for: Molecular Description of a Novel Orientia Species Causing Scrub Typhus in Chile
Source: Emerg Infect Dis. 2020 Sep;26(9):2148–56. doi: 10.3201/eid2609.200918 (PMC7454112; doi:10.3201/eid2609.200918)
Supplement: Appendix — Additional information for molecular description of a novel Orientia species causing scrub typhus in Chile. [file 20-0918-Techapp-s1.pdf]

Article DOI: https://doi.org/10.3201/eid2609.200918

Molecular Description of a Novel *Orientia* Species Causing Scrub Typhus in Chile

Appendix

Appendix Table 1. Primers and amplification conditions of semi-nested PCR assays

Target	Primer ID	Sequence (5′-3′)	Annealing temp.	Ref.
16S rRNA (*rrs*)	16sO79F*	ATTAATGCTGAGCTTGCTTAGCAT	51°C	(*1*)
	16sOR1198R*,†	TTTCCTATAGTTCCCGGCATT	51°C	(*1*)
	16s155F†	TCAGTACGGAATAACWTTTAGAAATAA	51°C	(*2*)
47-kDa (*htrA*)	Otr47_263F*	GTGCTAAGAAARGATGATACTTC	51°C	(*3*)
	Otr47_1133R*,†	ACATTTAACATACCACGACGAAT	51°C	(*3*)
	Otr47F†	TAAAGGTTAAGTTTATGAAAAAGGCATTT	51°C	(*4*)
56-kDa (*tsa*)	Otr56_498F*	AATTAGTTTAGAATGGTTACCAC	54°C	(*4*)
	r56_1459R*,†	TCTGTATCTGTTCGACAGATGCACTATTA	54°C	(*5*)
	Otr56_585F†	GAATGTCTGCGTTGTCGTTGC	54°C	(*4*)

*PCR.
†Semi-nested PCR.

Appendix Table 2. Identity matrix of rrs (upper right) and htrA (lower left). Percentage of pairwise identity of sequences and rrs/htrA genotypes of 18 Orientia isolates from Chile

Genotypes	BM 2016-I‡	LC 2016-I‡	MS 2016-M*‡	IS 2017-M‡	NV 2017-I‡	AE 2018-M‡	FC 2018-M‡	AF 2018-M‡	EC 2018-M‡	VP 2018-M‡	SH 2018-M§	GM 2019-I*¶	JC 2019-I¶	CC 2019-M¶	CV 2019-M¶	MA 2019-M§	MV 2019-M§	SG 2019-M*§
BM2016-I#		100	100	100	100	100	100	100	100	100	99.8	99.7	99.7	99.7	99.7	99.8	99.8	99.8
LC2016-I#	100		100	100	100	100	100	100	100	100	99.8	99.7	99.7	99.7	99.7	99.8	99.8	99.8
MS2016-M#	100	100		100	100	100	100	100	100	100	99.8	99.7	99.7	99.7	99.7	99.8	99.8	99.8
IS2017-M#	100	100	100		100	100	100	100	100	100	99.8	99.7	99.7	99.7	99.7	99.8	99.8	99.8
NV2017-I#	100	100	100	100		100	100	100	100	100	99.8	99.7	99.7	99.7	99.7	99.8	99.8	99.8
AE2018-M#	100	100	100	100	100		100	100	100	100	99.8	99.7	99.7	99.7	99.7	99.8	99.8	99.8
FC2018-M#	100	100	100	100	100	100		100	100	100	99.8	99.7	99.7	99.7	99.7	99.8	99.8	99.8
AF2018-M#	100	100	100	100	100	100	100		100	100	99.8	99.7	99.7	99.7	99.7	99.8	99.8	99.8
EC2018-M#	100	100	100	100	100	100	100	100		100	99.8	99.7	99.7	99.7	99.7	99.8	99.8	99.8
VP2018-M*#	100	100	100	100	100	100	100	100	100		99.8	99.7	99.7	99.7	99.7	99.8	99.8	99.8
SH2018-M	NA	NA	NA	NA	NA	NA	NA	NA	NA	NA		99.8	99.8	99.8	99.8	100	100	100
GM2019-I#	100	100	100	100	100	100	100	100	100	100	NA		100	100	100	99.8	99.8	99.8
JC2019-I††	99.8	99.8	99.8	99.8	99.8	99.8	99.8	99.8	99.8	99.8	NA	99.8		100	100	99.8	99.8	99.8
CC2019-M††	99.8	99.8	99.8	99.8	99.8	99.8	99.8	99.8	99.8	99.8	NA	99.8	100		100	99.8	99.8	99.8
CV2019-M††	99.8	99.8	99.8	99.8	99.8	99.8	99.8	99.8	99.8	99.8	NA	99.8	100	100		99.8	99.8	99.8
MA2019-M††	99.8	99.8	99.8	99.8	99.8	99.8	99.8	99.8	99.8	99.8	NA	99.8	100	100	100		100	100
MV2019-M††	99.8	99.8	99.8	99.8	99.8	99.8	99.8	99.8	99.8	99.8	NA	99.8	100	100	100	100		100
SG2019-M*††	99.8	99.8	99.8	99.8	99.8	99.8	99.8	99.8	99.8	99.8	NA	99.8	100	100	100	100	100	

NA, not amplified
*Deposited at GenBank
‡*rrs* 1 genotype
§*rrs* 2 genotype
¶*rrs* 3 genotype
#*htrA* a genotype
††*htrA* b genotype

References

1. Weitzel T, Abarca K, Martínez-Valdebenito C, Acosta-Jamett G, Jiang J, Richards AL. Scrub typhus in continental Chile, 2016–2018. Emerg Infect Dis. 2019;25:1214–7. PubMed https://doi.org/10.3201/eid2506.181860

2. Maina AN, Klein TA, Kim HC, Chong ST, Yang Y, Mullins K, et al. Molecular characterization of novel mosquito-borne *Rickettsia* spp. from mosquitoes collected at the Demilitarized Zone of the Republic of Korea. PLoS One. 2017;12:e0188327. PubMed https://doi.org/10.1371/journal.pone.0188327

3. Jiang J, Paris DH, Blacksell SD, Aukkanit N, Newton PN, Phetsouvanh R, et al. Diversity of the 47-kD HtrA nucleic acid and translated amino acid sequences from 17 recent human isolates of *Orientia*. Vector Borne Zoonotic Dis. 2013;13:367–75. PubMed https://doi.org/10.1089/vbz.2012.1112

4. Weitzel T, Aylwin M, Martínez-Valdebenito C, Jiang J, Munita JM, Thompson L, et al. Imported scrub typhus: first case in South America and review of the literature. Trop Dis Travel Med Vaccines. 2018;4:10. PubMed https://doi.org/10.1186/s40794-018-0070-8

5. Trung NV, Hoi LT, Cuong DD, Ha DT, Hoa TM, Lien VN, et al. Analysis of the 56-kDa type specific antigen gene of *Orientia tsutsugamushi* from northern Vietnam. PLoS One. 2019;14:e0221588. PubMed https://doi.org/10.1371/journal.pone.0221588
